# Quality of life in children and adolescents with hemophilia A: A systematic review and meta-analysis

**DOI:** 10.1016/j.rpth.2022.100008

**Published:** 2022-12-09

**Authors:** André Ferreira Azeredo-da-Silva, Bruna Stella Zanotto, Yukie Sato Kuwabara, Verónica Elizabeth Mata

**Affiliations:** 1 HTAnalyze Consulting and Training, Porto Alegre, Brazil; 2 National Institute for Health Technology Assessment (Instituto de Avaliacao de Tecnologias da Saude/Instituto Nacional de Ciencia e Tecnologia), Porto Alegre, Brazil; 3 F. Hoffmann-La Roche, São Paulo, Brazil

**Keywords:** adolescent, child, hemophilia A, quality of life, systematic review

## Abstract

**Background:**

Various instruments have been used to assess health-related quality of life (HRQoL) in children and adolescents with hemophilia A.

**Objective:**

We systematically reviewed the literature to summarize HRQoL measurement instruments and outcomes in this population.

**Methods:**

MEDLINE, Embase, Cochrane CENTRAL, and LILACS databases were searched. Studies published from 2010 to 2021, reporting HRQoL assessed by generic or hemophilia-specific instruments in individuals aged 0 to 18 years were included. Two independent reviewers performed screening, selection, and data abstraction. Data were meta-analyzed using the generic inverse variance method with the random-effects model for single-arm studies reporting instrument-specific mean total HRQoL scores. Prespecified subgroup meta-analyses were performed. Heterogeneity among studies was assessed using the *I*^*2*^ statistic.

**Results:**

Six instruments were identified in 29 studies meeting the following inclusion criteria: 4 generic instruments (PedsQL [5 studies], EQ-5D-3L [3 studies], KIDSCREEN-52 [1 study], and KINDL [1 study]) and 2 hemophilia-specific instruments (Haemo-QoL [17 studies] and CHO-KLAT [3 studies]). The overall risk of bias was moderate to low. There was a substantial variability in the primary outcome (mean total HRQoL score) among studies using the same instrument (Haemo-QoL), with scores ranging from 24.10 to 89.58 on a scale from 0 to 100 (higher scores indicating higher HRQoL). Meta-regression with 14 studies using the Haemo-QoL questionnaire demonstrated that 79.34% (*R*^*2*^) of the observed 94.67% total heterogeneity (*I*^*2*^) was explained by the proportion of patients receiving effective prophylactic treatment.

**Conclusion:**

HRQoL assessment in young people with hemophilia A is heterogeneous and context specific. The proportion of patients on effective prophylactic treatment is positively correlated with HRQoL. The review protocol was registered prospectively with PROSPERO (CRD42021235453).

## Essentials

• A systematic review on the quality of life (QoL) of children with hemophilia A was lacking.
• We searched major databases for studies reporting QoL in children with hemophilia A.
• QoL for this population varied according to the access to preventive measures.
• Better QoL was related to a higher proportion of patients receiving preventive therapy.

## 1 Introduction

Hemophilia A is a congenital life-threatening disorder caused by an inherited deficiency of factor (F)VIII [1]. It is characterized by spontaneous and post-traumatic bleeding events into the joints, muscles, and soft tissues, which can lead to disability. The challenges experienced by children and adolescents with hemophilia A can seriously affect their quality of life (QoL) in a wide range of health, physical, social, and educational settings. Parental restriction of the child’s or adolescent’s activities to prevent trauma, frequent absences from school due to repeated bleeding episodes, and the accompanying pain or discomfort associated with a bleeding episode are factors that limit or interfere with physical functioning, social and intellectual development, and academic performance [2,3]. Children and adolescents with hemophilia also experience difficulties that include frequent hospital visits, frequent injections, and inability to fully participate in daily activities [[4], [5], [6]].

Health-related QoL (HRQoL) is a multidimensional construct that includes a subjective assessment of the impact of health and illness on daily physical, social, cognitive, and emotional functioning [7]. Measurement of HRQoL is essential to fully understand the impact of a chronic disease and its treatment on clinical outcomes [1], and it should be included as an outcome in research and in the evaluation of treatment options for persons with hemophilia [8,9]. HRQoL questionnaires have long been used in adult populations and are gaining increasing acceptance among clinicians for use in children and adolescents [[10], [11], [12]]. Adult and pediatric HRQoL questionnaires can differ substantially in content and relevant dimensions, and the latter may also vary considerably with the age of the child [13].

Although age- and disease-specific HRQoL questionnaires have been developed [[14], [15], [16]], generic instruments have been most commonly used to assess HRQoL in patients with chronic conditions, such as the 36-item Short Form Health Survey for adults and the Pediatric Quality of Life Inventory (PedsQL) for children and adolescents [9,17,18]. Therefore, the challenge remains as to how to choose the best assessment tool for a particular chronic condition [1,5], and systematic reviews can be a useful starting point for identifying these tools [19].

In hemophilia, specifically, several systematic reviews have been published on the health and patient-reported outcome measures [11,[19], [20], [21]], but most of the studies are population specific and do not report the HRQoL measurement properties in children and adolescents [5]. Because clinical evaluation may not be sufficient to adequately characterize hemophilia A-related morbidity in children and adolescents, identifying tools that capture these features is important to guide HRQoL assessment and therapeutic care programs in the pediatric population with hemophilia A.

In the present systematic review, we aimed to identify and investigate existing instruments used to assess HRQoL in children and adolescents with hemophilia A and to summarize available QoL assessments.

## 2 Methods

We developed this systematic review according to the Preferred Reporting Items for Systematic Reviews and Meta-Analyses [22] statement and the methods for systematic reviews proposed by the Cochrane Collaboration [23]. The review protocol was registered prospectively with PROSPERO (CRD42021235453).

### 2.1 Search strategy

We searched MEDLINE (via PubMed), Embase, Cochrane Central Register of Controlled Trials (Cochrane CENTRAL), and Latin American and Caribbean Health Sciences Literature (LILACS via Virtual Health Library) databases for articles published from 2010 to February 2, 2021, by individually using the following keywords and search terms, including indexed terms (MeSH and EMTREE), subject indices, and synonyms, or by combining them with Boolean operators (“AND” and “OR”): “Hemophilia A,” “Congenital Hemophilia A,” “Classic Hemophilia,” “Factor VIII Deficiency, “Quality-Adjusted Life Years,” “Quality of Life,” “utility,” and “disability adjusted life year.” We did not include terms related to the intervention or study design to increase the search sensitivity. We set no language restrictions, but we only considered articles in the English, Portuguese, and Spanish for inclusion. We also hand searched the reference lists of all articles included in this review and of all reviews published on the subject for additional studies. Search strategy is provided in Supplementary Table 1. We cross-checked the results of the database searches to locate and eliminate duplicate entries.

### 2.2 Eligibility criteria and study selection

Studies eligible for inclusion in this review were randomized controlled trials and observational studies (cohort, cross-sectional, case control, or case series) published in English, Portuguese, or Spanish that recruited a minimum of 30 patients aged <18 years with hemophilia A and reported on the results of HRQoL assessment or condition-specific QoL assessment or utility/disutility assessment. Conference abstracts were considered for inclusion if they provided sufficient information about the primary outcome (mean total HRQoL score). There were no restrictions regarding the intervention used. Studies with or without a comparison group were also eligible for inclusion. We excluded the following study designs: guidelines, editorials, book chapters, commentaries, letters, notes, and study protocols.

We limited the review scope to persons with hemophilia A because this is the most prevalent form of hemophilia. Furthermore, restriction to 1 type of disease is likely to reduce the expected heterogeneity among studies.

The primary outcome of this review was the instrument-specific mean total HRQoL score of persons with hemophilia A, which was evaluated according to the study design and intervention. Secondary outcomes included the instrument used and the instrument-specific mean domain scores associated with better and worse HRQoL in a particular study.

### 2.3 Data extraction process

After removing duplicates, 2 reviewers (A.F.A.-d.-S. and B.S.Z.) independently screened the titles and abstracts of the retrieved articles and then assessed the full text of potentially eligible or uncertain studies for selection on the basis of our eligibility criteria. Any disagreements between reviewers were resolved by consensus or by a third independent reviewer (V.M.). In the presence of multiple reports of the same population, only the study with the largest sample size was selected.

For studies meeting eligibility, 2 reviewers (A.F.A.-d.-S. and B.S.Z.) independently extracted data using a standardized Excel spreadsheet (Microsoft Corporation). Disagreements were resolved by consensus or by a third independent reviewer (V.E.M.). Extracted data included the number of participants, sample characteristics, names and characteristics of the instruments used to measure QoL, comparison groups (when available), intervention protocols, and results of the QoL assessment.

### 2.4 Risk of bias assessment

Two reviewers (A.F.A.-d.-S. and B.S.Z.) independently assessed the risk of bias of included studies. Treatment-comparative cohort and case-control studies were assessed with the original Newcastle-Ottawa Scale (NOS), which contains 8 questions and assesses methodological quality using a star rating system that allocates a maximum of 9 stars across 3 categories: selection, comparability of the groups, and ascertainment of outcome (cohort studies) or exposure (case-control studies) [24]. Treatment-comparative cross-sectional studies were assessed with the NOS adapted for cross-sectional studies, which uses a similar star rating system (maximum of 10 stars) [25]. Noncomparative cohort and cross-sectional studies were assessed with the same scales with the exclusion of domains pertinent to comparative studies, resulting in a maximum of 6 and 8 stars for cohort and cross-sectional studies, respectively. Studies reaching 50% to 75% of the maximum number of stars for the given NOS were classified as having a moderate risk of bias, and those reaching 75% or more were classified as having a low risk of bias. We used the revised Cochrane risk of bias tool for randomized trials (RoB 2.0) to assess the randomized controlled trials [26].

### 2.5 Data analysis

We conducted qualitative and quantitative syntheses of the available evidence. For each included article, we collected data on which the QoL assessment tool was used and which QoL domains were assessed (eg, physical, mental, social life, hospitalization, school life, self-care, relationship with parents, and adherence to treatment, among others). Other relevant data extracted included study country, year of publication, study design, interventions used, number of patients, age of patients, ethnicity, parents’ level of education, household income, parents’ employment status, proportion of people with hemophilia A, proportion of patients with moderate-to-severe disease, proportion of patients receiving prophylactic and on-demand treatment, and proportion of patients with FVIII inhibitors. We reported the results stratified by study design and, whenever possible, according to the following prespecified subgroups: age group (0-7, 8-12, and 13-18 years), type of questionnaire, and FVIII inhibitor status.

We meta-analyzed the data using the generic inverse variance method with the random-effects model for single-arm studies reporting instrument-specific mean total HRQoL scores. For the main analysis, we organized the data into subgroups using the HRQoL instrument. Within each subgroup, we analyzed the instruments on a scale from 0 to 100 as reported in the original studies, with higher scores indicating better HRQoL. To match the overall scale of the meta-analysis, we transformed the results of 2 instruments: the QoL questionnaire for children with hemophilia (Haemo-QoL)—we analyzed the scores as 100 minus the reported score because, originally, higher scores indicated worse HRQoL in this specific instrument—and the three-level version of the EuroQol five-dimensional descriptive system for HRQoL states (EQ-5D-3L)—we analyzed the scores after multiplying them by 100 because, originally, the mean total HRQoL scores were reported on a scale from 0 to 1. We conducted other prespecified subgroup meta-analyses using the same methods.

For studies presenting direct group comparisons regarding the mean total HRQoL scores, we analyzed the standardized mean difference (SMD) of HRQoL scores between the groups being compared. We used an inverse variance random-effects model for meta-analysis.

We assessed heterogeneity among studies using the *I*^*2*^ statistic, where scores > 75% indicated high heterogeneity. We performed a meta-regression analysis for prespecified quantitative covariates whenever an unexplained high heterogeneity was found. We assessed the potential for publication bias by visually inspecting the funnel plots and using the Begg’s rank correlation test.

## 3 Results

**Figure 1:**
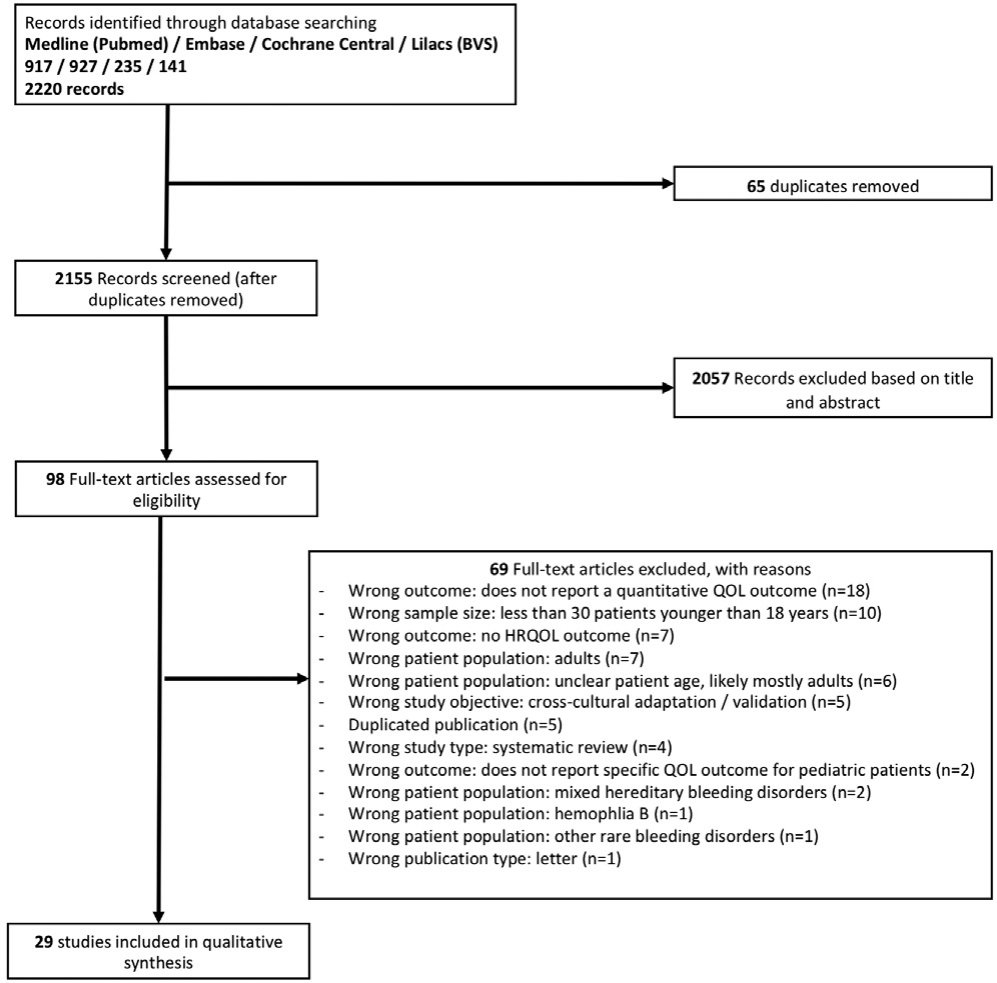
Flow diagram of the study selection process. BVS, biblioteva virtual de saude (virtual healthcare library); QOL, quality of life; HRQOL, health-related quality of life.

The initial search yielded 2220 records, of which 2155 remained after adjusting for duplicates. After screening the titles and abstracts, 98 studies were retrieved for full text reading, of which 29 met the inclusion criteria for the present review. Figure 1 shows the flow diagram of the study selection process.

Table 1 [6,[27], [28], [29], [30], [31], [32], [33], [34], [35], [36], [37], [38], [39], [40], [41], [42], [43], [44], [45], [46], [47], [48], [49], [50], [51], [52], [53], [54]] provides a brief description of each included study and an outline of the characteristics of each study population. The 29 included studies reported on 6 distinct instruments that were used to measure HRQoL in 2346 children and adolescents with hemophilia A. All articles were published from January 2010 to February 2021. Two studies used 2 different HRQoL questionnaires on their study sample; all other studies used only 1 questionnaire each [31,54].

**Table 1:** Characteristics of included studies and patients.

Author/year/country/publication type	Study design	N < 18 y and age range	Ethnicity and sociocultural or socioeconomic characteristics	Proportion of hemophilia A	Proportion of severe and moderate	Proportion of patients with inhibitors	Treatment type and proportion	QoL assessment tool
Ursu et al. [27]/2019/Romania/Conference abstract	Cross-sectional, no treatment comparison	37 “children”	Not reported	100%	Not reported	5.0% current	FVIII Proportion on prophylaxis not reported	EQ-5D/EuroQol VAS
Wu et al. [28]/2018/United States/Conference abstract	Cross-sectional, no treatment comparison	35 6-17 y	Not reported	Not reported	Not reported	Not reported	FVIII Proportion on prophylaxis not reported	PedsQL
Xu et al. [29]/2014/China/Conference abstract	Cross-sectional, no treatment comparison	37 of 132 patients had their QoL assessed 7-17.9 y	Not reported	87.1%	78.4%	Not reported	^a^FVIII: 91.0% on-demand, 9.0% prophylaxis (>6 mos).	CHO-KLAT
Limperg et al. [30]/2018/The Netherlands/Peer-reviewed journal article	Cross-sectional, no treatment comparison	87 patients 0-5 y: 27 6-7 y: 12 8-12 y: 20 13-18 y: 28	Parents’ level of education: 46.5% high level, 45.5% intermediate level, and 8.0% low level. 67.0% of parents reported having a paid employment.	81.6%	44.0%	Not reported	FVIII Proportion on prophylaxis not reported	TAPQOL^b^ for patients aged 0-5 y; PedsQL for patients aged 6-18 y.
Baek et al. [31]/2020/South Korea/Peer-reviewed journal article	Cross-sectional, no treatment comparison	100 of 605 persons with hemophilia 8-12 y: 47 13-16 y: 53	Not reported	83.6%^a^	100%	3.8% current^a^ 17.0% past or current^a^	^a^FVIII: 49.3% on-demand, 50.7% prophylaxis.	EQ-5D-3L (all patients). Haemo-QoL Long Form Kids II (8-12 y) and III (13-16 y).
Castro et al. [32]/2019/France, Germany, Italy, Spain, and United Kingdom/Conference abstract	Cross-sectional, no treatment comparison	171 Children and adolescents aged ≤17 y	Not reported	100%	100%	11.7% current	FVIII continuous prophylaxis in 30.0% for inhibitors, 70.0% for severe noninhibitors, and 45.0% for moderate noninhibitors.	EQ-5D-3L
Das et al. [33]/2019/India/Peer-reviewed journal article	Cross-sectional, no treatment comparison	201 4-16 y	Distribution according to social categories (castes): 61.7% general category, 13.9% scheduled castes, 4.5% scheduled tribes, and 19.9% other backward classes.	86.1%	78.6%	Not reported	FVIII Proportion on prophylaxis not reported	Haemo-QoL Long Form Kids II (8-12 y) and III (13-16 y).
Dekoven et al. [34]/2013/United States/Peer-reviewed journal article	Cross-sectional, no treatment comparison	52 4-16 y	Not reported	88.7%	Not reported	100% current	FVIII: 57.7% on-demand, 42.3% prophylaxis.^a^	Haemo-QoL Long Form Kids II (8-12 y) and III (13-16 y).
Dsouza et al. [35]/2020/India/Peer-reviewed journal article	Cross-sectional, no treatment comparison	107 6-16 y	Distribution according to socioeconomic class: 18.7% poor class, 67.3% lower middle class, and 14.0% upper middle class.	89.7%	100%	0.0%	FVIII Proportion on prophylaxis not reported	Haemo-QoL Long Form Kids II (8-12 y) and III (13-16 y).
Kearney et al. [36]/2019/Germany/Peer-reviewed journal article	Comparative before and after cohort (FVIII vs turoctocog alfa) Follow-up: - 6.0 mos (Pathfinder 5 cohort aged 4-12 y) - 18.0 mos (Pathfinder 2 cohort aged ≥12 y)	45 4-16 y	Not reported	100%	100% severe	0.0%	Before and after study: Baseline: 100% intravenous FVIII infusions^c^ Main study phase: 100% turoctocog alfa pegol (pegylated recombinant human factor VIII); 50.0% previous	Haemo-QoL I (4-7 y), II (8-12 y), and III (13-16 y).
Khair et al. [37]/2017/United Kingdom/Peer-reviewed journal article	Cross-sectional, no treatment comparison	127 8-17 y	Not reported	89.7%	100% severe	7.1% current 19.7% past or current	FVIII: 2.4% on-demand, 97.6% prophylaxis.	Haemo-QoL Short Form II (8-12 y).
Mancuso et al. [38] (HAVEN 2)/2020/United States/Peer-reviewed journal article	Comparative before and after cohort (FVIII vs emicizumab) Follow-up: 11.4 mos	34 8-12 y	Distribution according to ethnicity: 61.4% white, 14.8% Asian, 13.6% black, 2.3% mixed race, and 8.0% unknown.	100%	96.6% severe 1.1% moderate	100% current	Previous regimen (BPA): 75.0% prophylaxis, 25.0% episodic. Main study phase: 100% emicizumab subcutaneous loading dose of 3.0 mg/kg for 4 weeks, followed by a maintenance dose of either 1.5 mg/kg/week, 3.0 mg/kg/every other week, or 6.0 mg/kg/every 4 weeks.	Haemo-QoL Short Form II (8-12 y).
Mercan et al. [6]/2010/Turkey/Peer-reviewed journal article	Cross-sectional, no treatment comparison	39 4-16 y	Not reported	74%	92% severe	0%	FVIII: 80.0% on-demand, 20.0% prophylaxis.	Haemo-QoL I (4-7 y), II (8-12 y), and III (13-16 y).
Mousavi et al. [39]/2019/Afghanistan/Peer-reviewed journal article	Cross-sectional, no treatment comparison	65 8-16 y	Most patients were born to low-income (95.0%) and illiterate families (>50.0%).	100%	80.0% severe 16.9% moderate	Not reported	FVIII Proportion on prophylaxis not reported	Haemo-QoL Short Form II (8-12 y) and III (13-16 y).
Poon et al. [40]/2012 (POON 2012)/United States/Conference abstract	Cross-sectional, no treatment comparison	61 2-18 y	Not reported	≥ 86.0%	Not reported	13.0%	FVIII: 87.0% prophylaxis.	PedsQL
Poon et al. [41]/2014 (POON 2014 QoL Cohort)/United States/Peer-reviewed journal article	Cohort, no treatment comparison Follow-up: 6.0 mos	125 2-17 y	68.0% of the study children were of white/non-Hispanic ethnicity. 73.6% of parents had a part-time or full-time employment.	100%	67.2% severe or moderate	Not reported	FVIII: 42.2% prophylaxis.	PedsQL
Poon et al. [42]/2012 (HUGS-Va)/United States/Peer-reviewed journal article	Cross-sectional, no treatment comparison	164 2-18 y	Not reported	100%	66.5% severe 12.0% moderate	0.0%	FVIII: 55.5% prophylaxis.	PedsQL
Santagostino et al. [43]/2014/Canada/Peer-reviewed journal article	Comparative before and after cohort (FVIII vs turoctocog alfa) Follow-up: 4.5-6.0 mos	64 2-18 y	Not reported	100%	100% severe	0.0%	FVIII infusion (baseline), type of regimen by age group: 4-7 y: 32.0% on-demand, 68.0% prophylaxis. 8-12 y: 23.8% on-demand, 66.7% prophylaxis, and 9.5% mixed regimen. 13-18 y: 44.4% on-demand, 22.2% prophylaxis, and 33.3% mixed regimen.	Haemo-QoL I (4-7 y), II (8-12 y), and III (13-16 y).
Taha and Hassan [44]/2014/Iraq/Peer-reviewed journal article	Cross-sectional, no treatment comparison	45 4-16 y	All included patients were from the peripheries of Basra (Southern Iraq) and belonged to families of low socioeconomic status.	88.9%	46.6% severe 26.7% moderate	22.2% current	FVIII: 100% on-demand.	Haemo-QoL I (4-7 y), II (8-12 y), and III (13-16 y).
Tang et al. [45]/2018/China/Peer-reviewed journal article	Cross-sectional, no treatment comparison	133 4-18 y	Not reported	90.7%	36.1% severe 52.0% moderate	0.0%	FVIII: 55.8% on-demand, 44.2% prophylaxis.	CHO-KLAT
Tantawy et al. [46]/2011/Egypt/Peer-reviewed journal article	Cross-sectional, no treatment comparison	60 4-16 y	Not reported	100%	100% severe	5.0%	FVIII: 68.0% on-demand, 32.0% prophylaxis.	Haemo-QoL I (4-7 y), II (8-12 y), and III (13-16 y).
Furuichi et al. [47]/2020/Japan/Peer-reviewed journal article	Cross-sectional, no treatment comparison	36 8-18 y	38.9% of social support weakness among persons with hemophilia as assessed by the Oslo 3-item social support scale.	86.1%	77.8% severe 19.4% moderate	8.3%	FVIII: 5.6% on-demand, 94.4% prophylaxis.	J-KIDSCREEN-52
Broderick et al. [48]/2010/Australia/Peer-reviewed journal article	Cross-sectional, no treatment comparison	41 of 45 had hemophilia A or B 6-17 y	Not reported	Not reported	48.0% severe 25.0% moderate	Not reported	FVIII: 30.0% on-demand, 70.0% prophylaxis.	Haemo-QoL I (4-7 y), II (8-12 y), and III (13-16 y).
Zhang et al. [49]/2019/China/Peer-reviewed journal article	Cohort, no treatment comparison Follow-up: 4.0 y	42 5.5 ± 4.6 y	Not reported	88.1%	33.0% severe 67.1% moderate	0%	FVIII: 59.5% on-demand, 40.5% prophylaxis.	CHO-KLAT
Gringeri et al. [50]/2011/Italy/Peer-reviewed journal article	Randomized controlled trial Follow-up: median of 6.9 y	Full randomized sample: 45 (effective sample = 40) Sample with QoL assessment at the end of follow-up: - Prophylaxis group: 21 of 23 patients - Episodic treatment: 19 of 22 patients Enrollment age: 1-7 y Prophylaxis group: 4.1 ± 2.2 y Episodic treatment: 4.1 ± 1.8 y	Not reported	100%	100% severe	0.0%	FVIII: 50.0% on-demand (episodic), 50.0% prophylaxis.	Haemo-QoL I (4-7 y), II (8-12 y), and III (13-16 y).
Lock et al. [51]/2016/The Netherlands/Peer-reviewed journal article	Cohort, no treatment comparison Follow-up: 1.8 y	46 1-18 y (9.4 ± 4.2)	Patients’ mothers’ highest level of education was as follows: 13.0% low level, 62.0% intermediate level, and 25.0% high level. 82.0% of mothers had a paid employment when the study was conducted.	78.0%	91.0% severe 7.0% moderate	0.0%	FVIII: 100% prophylaxis.	Haemo-QoL I (4-7 y), II (8-12 y), and III (13-16 y).
Papagianni et al. [52]/2016/Greece/Conference abstract	Cross-sectional, no treatment comparison	45 4-16 y (10.5 ± 4.3)	Not reported	86.7%	Not reported	Not reported	FVIII: 77.8% prophylaxis.	Haemo-QoL I (4-7 y), II (8-12 y), and III (13-16 y).
Hassab et al. [53]/2016/Egypt/Conference abstract	Cross-sectional, no treatment comparison	50 4-16 y, with hemophilic arthropathy	Not reported	72.0%	46.0% severe 54.0% moderate	Not reported	FVIII: 100% on-demand.	Haemo-QoL I (4-7 y), II (8-12 y), and III (13-16 y).
Khair et al. [54]/2012/United Kingdom/Peer-reviewed journal article	Cross-sectional, no treatment comparison	84 6-17 y	Not reported	91.7%	50.0% severe 22.6% moderate	6.0%	FVIII: 33.3% on-demand, 66.7% prophylaxis.	KINDL and Haemo-QoL I (4-7 y), II (8-12 y), and III (13-16 y).

BPA, bypassing agent; FVIII, coagulation factor VIII; QoL, quality of life; VAS, visual analog scale.

a Proportions from a larger sample that may include patients aged ≥18 y.

b Study publication does not present results for the TAPQOL applied to patients aged 0 to 5 y, but the authors report that there were no differences in HRQoL between boys with and without hemophilia as measured with the TAPQOL instrument.

c Distribution of regular FVIII intravenous infusions according to treatment regimen by age group [36]: 91.2% prophylaxis and 8.8% on-demand in the 4 to 7 y age group; 100% prophylaxis in the 8 to 12 y age group; and 85.1% prophylaxis and 14.9% on-demand in the 13 to 16 y age group.

Ten studies measured HRQoL with one of the following 4 generic instruments: PedsQL (5 studies) [28,30,[40], [41], [42]], EQ-5D-3L (3 studies) [27,31,32], KIDSCREEN-52 (1 study) [47], and KINDL (1 study) [54]. Twenty studies measured HRQoL with one of the following hemophilia-specific pediatric instruments: Haemo-QoL (18 studies) [6,31,[33], [34], [35], [36],^38^,^39^,^43^,^44^,^46^,^48^,[50], [51], [52], [53],^55^] and the Canadian Hemophilia Outcomes—Kids’ Life Assessment Tool (CHO-KLAT) (3 studies) [29,45,54].

Regarding patient characteristics, 13 studies reported HRQoL outcomes for patients aged 0 to 7 years, 16 for patients aged 8 to 12 years, and 15 for patients aged 13 to 18 years. The remaining studies reported HRQoL outcomes for children and adolescents in general (0-18 years of age). Ethnicity and sociocultural or socioeconomic information were reported in 9 studies (Table 1) [30,33,35,38,39,41,44,47,51].

Hemophilia A corresponded to 90.0% (median) of the overall patient population (range, 72.0%-100%), and 97.0% (median) were reported as moderate or severe hemophilia cases (range, 44.0%-100%). FVIII inhibitor status was reported in 20 of 29 studies. Two studies included only persons with hemophilia A with inhibitors [34,38], whereas 9 studies included only patients without inhibitors [6,35,36,42,43,45,50,51,54]. The remaining studies reported a median of 6.5% of patients with FVIII inhibitors (range, 3.3%-22.0%).

The overall risk of bias was assessed as moderate to low. The 22 noncomparative cross-sectional studies had a median of 6 of 8 stars (range, 1-7 stars) on the corresponding adapted NOS. The 3 cohort studies with no treatment comparison were classified as having a low risk of bias (6 of 6 stars) on the corresponding adapted NOS. Three treatment-comparative cohort studies had a median of 5 of 9 stars on the original NOS for cohort studies. The only randomized controlled trial, conducted by Gringeri et al. [50], had an overall low risk of bias on the Cochrane RoB 2.0 tool (“some concerns” in the domain “deviation from the intended intervention”). The results of the risk of bias assessment according to the study design are presented in Table 2 [6,[27], [28], [29], [30], [31], [32], [33], [34], [35], [36], [37], [38], [39], [40], [41], [42], [43], [44], [45], [46], [47], [48], [49], [50], [51], [52], [53], [54]].

**Table 2:** Risk of bias assessment according to the study design.

Author/year/country/publication type	Study design	RoB assessment instrument	Assessment result
Ursu et al. [27] /2019/ Romania/Conference abstract	Cross-sectional, no treatment comparison	NOS cross-sectional adaptation^†^	1 of 8 possible score points
Wu et al. [28]/2018/United States/Conference abstract	Cross-sectional, no treatment comparison	NOS cross-sectional adaptation^†^	5 of 8 possible score points
Xu et al. [29]/2014/China/Conference abstract^a^	Cross-sectional, no treatment comparison	NOS cross-sectional adaptation^b^	4 of 8 possible score points
Limperg et al. [30]/2018/The Netherlands/Peer-reviewed journal article	Cross-sectional, no treatment comparison	NOS cross-sectional adaptation^b^	7 of 8 possible score points
Baek et al. [31]/2020/South Korea/Peer-reviewed journal article	Cross-sectional, no treatment comparison	NOS cross-sectional adaptation^b^	6 of 8 possible score points
Castro et al. [32]/2019/France, Germany, Italy, Spain, and United Kingdom/Conference abstract	Cross-sectional, no treatment comparison	NOS cross-sectional adaptation^b^	5 of 8 possible score points
Das et al. [33]/2019/India/Peer-reviewed journal article	Cross-sectional, no treatment comparison	NOS cross-sectional adaptation^b^	5 of 8 possible score points
Dekoven et al. [34]/2013/United States/Peer-reviewed journal article	Cross-sectional, no treatment comparison	NOS cross-sectional adaptation^b^	7 of 8 possible score points
Dsouza et al. [35]/2020/India/Peer-reviewed journal article	Cross-sectional, no treatment comparison	NOS cross-sectional adaptation^b^	7 of 8 possible score points
Kearney et al. [36]/2019/Germany/Peer-reviewed journal article	Comparative before and after cohort	NOS for cohort studies^b^	5 of 9 possible score points
Khair et al. [37]/2017/United Kingdom/Peer-reviewed journal article	Cross-sectional, no treatment comparison	NOS cross-sectional adaptation^b^	7 of 8 possible score points
Mancuso et al. [38] (HAVEN 2)/2020/United States/Peer-reviewed journal article	Comparative before and after cohort	NOS for cohort studies^b^	6 of 9 possible score points
Mercan et al. [6]/2010/Turkey/Peer-reviewed journal article	Cross-sectional, no treatment comparison	NOS cross-sectional adaptation^b^	7 of 8 possible score points
Mousavi et al. [39]/2019/Afghanistan/Peer-reviewed journal article	Cross-sectional, no treatment comparison	NOS cross-sectional adaptation^b^	6 of 8 possible score points
Poon et al. [40]/2012 (POON 2012)/ United States/Conference abstract	Cross-sectional, no treatment comparison	NOS cross-sectional adaptation^b^	5 of 8 possible score points
Poon et al. [41]/2014 (POON 2014 QoL Cohort)/ United States/Peer-reviewed journal article	Cohort, no treatment comparison	NOS for cohort studies^b^	6 of 6 possible score points
Poon et al. [42]/2012 (HUGS-Va)/ United States/Peer-reviewed journal article	Cross-sectional, no treatment comparison (regarding QoL outcome)	NOS cross-sectional adaptation^b^	6 of 8 possible score points
Santagostino et al. [43]/2014/Canada/Peer-reviewed journal article	Comparative before and after cohort	NOS for cohort studies^b^	5 of 9 possible score points
Taha and Hassan [44]/2014/Iraq/Peer-reviewed journal article	Cross-sectional, no treatment comparison	NOS cross-sectional adaptation^b^	7 of 8 possible score points
Tang et al. [45]/2018/China/Peer-reviewed journal article	Cross-sectional, no treatment comparison	NOS cross-sectional adaptation^b^	7 of 8 possible score points
Tantawy et al. [46]/2011/Egypt/Peer-reviewed journal article	Cross-sectional, no treatment comparison	NOS cross-sectional adaptation^b^	6 of 8 possible score points
Furuichi et al. [47]/2020/Japan/Peer-reviewed journal article	Cross-sectional, no treatment comparison	NOS cross-sectional adaptation^b^	5 of 8 possible score points
Broderick et al. [48]/2010/Australia/Peer-reviewed journal article	Cross-sectional, no treatment comparison	NOS cross-sectional adaptation^b^	4 of 8 possible score points
Zhang et al. [49]/2019/China/Peer-reviewed journal article	Cohort, no treatment comparison	NOS for cohort studies^b^	6 of 6 possible score points
Gringeri et al. [50]/2011/Italy/Peer-reviewed journal article	Randomized controlled trial	Cochrane RoB 2.0 tool	Low overall risk of bias
Lock et al. [51]/2016/The Netherlands/Peer-reviewed journal article	Cohort, no treatment comparison	NOS for cohort studies^b^	6 of 6 possible score points
Papagianni et al. [52]/2016/Greece/Conference abstract	Cross-sectional, no treatment comparison	NOS cross-sectional adaptation^b^	4 of 8 possible score points
Hassab et al. [53]/2016/Egypt/Conference abstract	Cross-sectional, no treatment comparison	NOS cross-sectional adaptation^b^	4 of 8 possible score points
Khair et al. [54]/2012/United Kingdom/Peer-reviewed journal article	Cross-sectional, no treatment comparison	NOS cross-sectional adaptation^b^	6 of 8 possible score points

NOS, Newcastle-Ottawa Scale; RoB, risk of bias.

a Abstract-based RoB assessment is limited. Missing information assumed to increase RoB.

b NOS for comparative cohort studies (maximum 9 points) and adaptation for comparative cross-sectional studies (maximum 10 points); domains pertinent to comparative studies were not included in the assessment of single-arm studies, resulting in maximum values of 6 and 8 points for the assessments of noncomparative cohort and cross-sectional studies, respectively.

There was a substantial variability in the primary outcome (mean total HRQoL score) among studies that used the same instrument. Scores ranged from as low as 24.10 to as high as 89.58 on a scale from 0 to 100, where higher scores indicate better HRQoL. Table 3 [6,[27], [28], [29], [30], [31], [32], [33], [34], [35], [36], [37], [38], [39], [40], [41], [42], [43], [44], [45], [46], [47], [48], [49], [50], [51], [52], [53], [54]] shows the total HRQoL scores and a brief description of the main findings of each study.

**Table 3:** HRQoL outcomes.

ID	Author/year/country/publication type	QoL assessment tool	Respondents^a^	Mean overall score (± SD or range)^b^	Key HRQoL findings
1	Ursu et al. [27] 2019/Romania/Conference abstract	EuroQol VAS	Not reported	82.30 ± 20.90	Children scored higher in EuroQol VAS than adults (82.30 ± 20.90 vs 63.50 ± 14.10). Pain correlated significantly with age (r = 0.44, P = .003) and with Hemophilia Joint Health Score (r = 0.61, P < .001).
2	Wu et al. [28]/2018/United States/Conference abstract	PedsQL, children aged 6-17 y	Not reported	83.23 ± 20.02	Children had mean total PedsQL, physical health, and psychosocial health scores of 83.23 ± 20.02, 84.83 ± 24.69, and 82.43 ± 19.23, respectively. Multivariable regression models for the child sample showed that bleeding-related pain was the most important variable associated with lower physical health, whereas chronic pain and having public insurance were associated with reduced psychosocial health.
3	Xu et al. [29]/2014/China/Conference abstract	CHO-KLAT	Patient self-report and parent/caregiver report	55.32 ± 9.90	QoL as reported by both patients and parents was poor. The CHO-KLAT scores reported by patients (55.32 ± 9.90) and by parents (54.10 ± 9.63) were similar, with a correlation of 0.34 (P < .050).
4	Limperg et al. [30]/2018/The Netherlands/Peer-reviewed journal article	PedsQL	Patient self-report and parent/caregiver report	Age 6-7 y: 94.57 (range, 50-100) Age 8-12 y: 86.96 (range, 65-99) Age 13-18 y: 90.22 (range, 49-100)	Children (0-12 y) showed no significant impairment in HRQoL compared with healthy peers. Adolescent boys (13-18 y) with CBDs reported slightly better HRQoL on the total and emotional functioning scales than healthy peers (small-moderate effect sizes). In contrast, adolescent girls experienced worse HRQoL on the total, social functioning, and psychosocial health scales than healthy peers (moderate effect sizes). No differences were found in HRQoL between severity groups, but more problem behavior was observed in young boys (0-5 y) with severe hemophilia. Male sex, participation in sports, and school attendance were positively associated with HRQoL. Parental country of birth, type of treatment, and number of bleeds were not associated with HRQoL.
5	Baek et al. [31]/2020/South Korea/Peer-reviewed journal article	EQ-5D-3L (all patients) and EuroQol VAS Haemo-QoL Long Form Kids II (8-12 y) and III (13-16 y)	Patient self-report	Haemo-QoL Long Form Kids II (8-12 y): 26.44 ± 11.32 Haemo-QoL Long Form Kids III for the adolescent group (13-16 y): 28.88 ± 11.06	Mean EQ-5D-3L index score and EuroQol VAS score in all persons with hemophilia (including adults) were 0.68 ± 0.15 and 70.22 ± 19.75, respectively. Mean Haemo-QoL scores revealed significant differences by age group (children vs adolescent vs adult: 26.44 ± 11.30 vs 28.88 ± 11.10 vs 38.43 ± 17.70, respectively; P < .001). “Sports and leisure,” “Family planning,” and “View” in adults and “Perceived support,” “Friends,” and “Dealing” in children and adolescents were identified as the domains with the greatest HRQoL impairments. In a multivariable regression analysis, HRQoL was negatively associated with the presence of hemophilia-induced disability (β = 0.222, P < .000), bleeding events within the past 6 mos (β = 0.098, P = .010), and hemophilic arthropathy (β = 0.212, P < .000).
6	Castro et al. [32]/2019/France, Germany, Italy, Spain, and United Kingdom/Conference abstract	EQ-5D-3L	Patient self-report and parent/caregiver report	Patients without inhibitors: - overall: 0.66 ± 0.27 - moderate disease: 0.61 ± 0.36 - severe disease: 0.67 ± 0.24 Patients with inhibitors: - moderate or severe disease: 0.52 ± 0.34	Median EQ-5D index score was lower for patients with inhibitors than for those without inhibitors (0.57 vs 0.69, P = .040). Most patients reported some level of activity limitation (59.0%), pain (70.0%), and/or anxiety/depression (62.0%). Patients with inhibitors were significantly more likely to experience mobility impairment (60.0% vs 34.0%, P = .030), to be diagnosed with depression (16.0% vs 2.0%, P = .020), and to have severe/moderate pain (53.0% vs 19.0%, P < .010). Patients with moderate disease seem to have a burden on QoL that is not significantly different from severe patients.
7	Das et al. [33]/2019/India/Peer-reviewed journal article	Haemo-QoL Long Form Kids II (8-12 y) and III (13-16 y)	Patient self-report	Mean Haemo-QoL scores: Age 4-7 y: 43.92 ± 8.09 Age 8-12 y: 37.37 ± 8.62 Age 13-16 y: 32.79 ± 6.66 Age >16 y: 45.92 ± 6.30	“Physical health” in all 4 age groups, “View” in 8-12 y and 13-16 y, “Perceived support” in 8-12 y, “Others” in 13-16 y, “Dealing” in 8-12 y, and “Future” in 13-16 y dimension scores (transformed) showed a significant positive correlation with WFH joint scores in the Spearman correlation analysis.
8	Dekoven et al. [34]/2013/United States/Peer-reviewed journal article	Haemo-QoL Long Form Kids II (8-12 y) and III (13-16 y)	Parent/caregiver report	Mean Haemo-QoL scores: Age 4-7 y: 35.00 ± 16.10 Age 8-16 y: 33.80 ± 15.50	Among persons with hemophilia with inhibitors, HRQoL impairment increased with age. Caregiver burden also affected the perceived HRQoL of children with hemophilia with inhibitors. The HRQoL domains with the highest impairment by age group were as follows: 4-7 y, “Family” (mean = 55.60), “Treatment” (mean = 39.30), and “Friends” (mean = 38.10); 8-16 y, “Physical health” (mean = 50.80), “Sports and school” (mean = 42.10), and “Perceived support” (mean = 40.10).
9	Dsouza et al. [35]/2020/India/Peer-reviewed journal article	Haemo-QoL Long Form Kids II (8-12 y) and III (13-16 y)	Patient self-report	Mean Haemo-QoL scores: Age 6-7 y: 55.41 ± 14.48 Age 8-12 y: 49.62 ± 10.12 Age 13-16 y: 47.62 ± 9.97	The “Family” and “Other persons” domains were highly impaired in children aged 6-7 y. QoL was also impaired in “Family” and “Friends” compared with “Physical health” and “Feeling” domains in children aged 8-12 y.
10	Kearney et al. [36]/2019/Germany/Peer-reviewed journal article	Haemo-QoL I (4-7 y), II (8-12 y), and III (13-16 y)	Patient self-report and parent/caregiver report	Mean baseline Haemo-QoL scores: Age 4-7 y: 29.60 ± 17.90 Age 8-12 y: 21.70 ± 10.00 Age 13-16 y: 18.50 ± 9.60 Mean final (after treatment) Haemo-QoL scores: Age 4-7 y: 22.30 ± 12.40 Age 8-12 y: 17.90 ± 9.80 Age 13-16 y: 15.70 ± 9.40	Mean changes in the total child/adolescent-reported Haemo-QoL scores were −14.00 for ages 4-7 y, −3.60 for ages 8-11 y, and −0.10 for ages 13-16 y. Although most patients reported a relatively good baseline HRQoL when entering the respective trials, the HRQoL of patients was either maintained or further improved when treated with turoctocog alfa pegol.
11	Khair et al. [37]/2017/United Kingdom/Peer-reviewed journal	Haemo-QoL Short Form II (8-12 y)	Patient self-report	Mean total Haemo-QoL score: 22.81 ± 15.00	Children reported generally good HRQoL in the total score (mean = 22.81 ± 15.00), with the highest impairments in the domains “Friends” (mean = 28.18 ± 30.50) and “Sports and school” (mean = 26.14 ± 25.10).
12	Mancuso et al. [38] (HAVEN 2)/2020/United States/Peer-reviewed journal article	Haemo-QoL Short Form II (8-12 y)	Patient self-report and parent/caregiver report	Mean total Haemo-QoL scores: Baseline: 30.20 ± 14.90 Week 13: 24.80 ± 14.00 Week 25: 19.80 ± 13.50 Week 37: 19.00 ± 13.90 Week 49: 23.00 ± 13.90	In HAVEN 2 (n = 88), the median age was 6.5 y (range, 1-15 y), and 34 participants aged 8-11 y completed the Haemo-QoL Short Form II questionnaire. Mean baseline total score was 30.20 ± 14.90 (n = 30), indicating moderate impairment. With emicizumab, the mean score decreased by −9.62 (±7.73; n = 17) points to 23.00 (±13.93; n = 20) by week 49. The most improved domains were “Sports and school” and “Physical health.” Caregivers reported similar improvements. Prophylactic emicizumab was accompanied by substantial and sustained improvements in the HRQoL of children and adolescents with hemophilia A with FVIII inhibitors and their caregivers.
13	Mercan et al. [6]/2010/Turkey/Peer-reviewed journal article	Haemo-QoL I (4-7 y), II (8-12 y), and III (13-16 y)	Patient self-report	Mean Haemo-QoL scores: Age 4-7 y: 39.60 ± 15.00 Age 8-12 y: 39.90 ± 9.90 Age 13-16 y: 39.80 ± 16.70	Mean Haemo-QoL scores were 39.60 ± 15.00 for children/adolescents (n = 36) and 47.40 ± 14.10 for adults (n = 31). Mean total WFH joint scores were 1.83 ± 2.70 for ages 4-7 y, 4.90 ± 4.96 for ages 8-12 y, and 6.94 ± 6.15 for ages 13-16 y. They were more impaired in adults (16.23 ± 14.12). The results showed that the Turkish versions of the Haemo-QoL and Haem-A-QoL are reliable tools to measure QoL in children and adults with hemophilia.
14	Mousavi et al. [39]/2019/Afghanistan/Peer-reviewed journal article	Haemo-QoL Short Form II (8-12 y) and III (13-16 y)	Patient self-report	Mean Haemo-QoL score: 75.90 ± 17.40 (ages 8-16 y)	Mean patient age was 12.9 ± 3.9 y, and mean Haemo-QoL score was 75.90 ± 17.40. Patients had hemophilia A, mostly the severe type (80.0%). They were from low-income families (95.0%) with high illiteracy rates (>50.0%) and hemophilia family history (90.0%). Spearman test showed a significant correlation between age and QoL scores (r = 0.80, p = .020). One-way analysis of variance showed no significant difference in QoL scores between patients categorized based on hemophilia severity (p = .200, F = 1.30), family income (P = .900, F = 0.01), and parents’ level of education (P = .200 to .400, F = 0.82-1.30). “Family” and “Sports” were the most impaired QoL domains.
15	Poon et al. [40]/2012 (POON 2012)/ United States/Conference abstract	PedsQL	Parent/caregiver report	Mean total PedsQL scores: Inhibitors: 66.70 ± 9.90 Noninhibitors: 78.20 ± 15.30	Inhibitor patients had significantly lower total PedsQL (66.70 ± 9.90 vs 78.20 ± 15.30, P = .028) and emotional functioning scores (65.60 ± 12.10 vs 77.20 ± 19.60, P = .049) than noninhibitor patients. Among inhibitor patients, there were no notbable differences in PedsQL scores between prophylaxis and on-demand. Inhibitor patients also experienced significantly more joint bleeds (4.80 ± 3.10 vs 3.10 ± 4.70, P = .006) in the 12 mos before the survey and developed significantly more target joints (3.80 ± 0.50 vs 0.60 ± 0.80, P < .000). More noninhibitor patients (60.4%) reported a full range of motion than inhibitor patients (25.0%), although similar proportions of noninhibitors (90.6%) and inhibitors (87.5%) reported none or slight bodily pain that interfered with their activities.
16	Poon et al. [41]/2014 (POON 2014 QoL Cohort)/ United States/Peer-reviewed journal article	PedsQL	Parent/caregiver report	Mean total PedsQL scores not reported	Mean PedsQL psychosocial and physical functioning scores were 83.00 ± 15.70 and 88.00 ± 16.40, respectively, for the pediatric study population, with greater variation in physical functioning than in psychosocial functioning scores over time.
17	Santagostino et al. [43]/2014/Canada/Peer-reviewed journal article	Haemo-QoL I (4-7 y), II (8-12 y), and III (13-16 y)	Patient self-report and parent/caregiver report	Baseline total scores (child-completed version): Age 4-7 y: 30.00 ± 13.60 Age 8-12 y: 26.10 ± 8.90 Age 13-18 y: 31.40 ± 9.60	Mean change from baseline (FVIII vs turoctocog alfa) in all scores was described for 25 children aged 4-7 y, 21 children aged 8-12 y, and 18 adolescents aged 13-18 y overall and according to the treatment regimen received before the trial (on-demand, prophylaxis, or mixed). Mean changes in total Haemo-QoL score were 1.40 for children aged 4-7 y, 2.60 for children aged 8-12 y, 5.80 for adolescents, and 1.60 for adults. In parent-completed versions, mean changes in the total score were 6.00 for children aged 4-7 y, 4.70 for children aged 8-12 y, and 10.00 for adolescents. Patients receiving on-demand treatment before the trial showed greater improvement in HRQoL scores than patients already on prophylaxis.
18	Taha and Hassan [44]/2014/Iraq/Peer-reviewed journal article	Haemo-QoL I (4-7 y), II (8-12 y), and III (13-16 y)	Patient self-report and parent/caregiver report	Severe disease: 58.51 ± 3.62 Moderate disease: 37.55 ± 5.37 Mild disease: 35.47 ± 4.19	Severity of hemophilia adversely affected QoL. Among children with severe hemophilia, young children who had ≥5 joint bleeds during the last year had significant impairment in the total score and in several domain scores, including “Physical health,” “Feeling,” and “Treatment,” compared with children who had <5 joint bleeds.
19	Tang et al. [45]/2018/China/Peer-reviewed journal article	CHO-KLAT	Patient self-report and parent/caregiver report	On-demand: - Child CHOK-LAT (n = 90): 56.10 ± 12.72 - Parent CHOK-LAT (n = 90): 53.80 ± 12.39 Prophylaxis: - Child CHOK-LAT (n = 43): 59.80 ± 11.00 - Parent CHOK-LAT (n = 43): 53.70 ± 11.93	Child self-report CHO-KLAT scores were available for 171 boys aged ≥7 y and ranged from 24.20 to 85.30, with a mean of 57.60 (n = 171). Parent proxy-reported CHO-KLAT scores ranged from 25.00 to 88.70, with a mean of 55.10 (n = 269). HRQoL scores in Chinese boys with hemophilia were substantially lower than in Canadian and European boys with hemophilia.
20	Tantawy et al. [46]/2011/Egypt/Peer-reviewed journal article	Haemo-QoL I (4-7 y), II (8-12 y), and III (13-16 y)	Patient self-report	Mean Haemo-QoL scores: Age 4-7 y: 61.60 ± 17.20 Age 8-12 y: 58.00 ± 18.70 Age 13-16 y: 63.80 ± 18.20	HRQoL scores were widely above 50 for all dimensions, being highly significant in 3 dimensions (Physical health, Family, and Treatment) in different age groups. HRQoL was impaired in the “Physical health” domain for 2 groups and in the “Family” domain for the oldest group, whereas the youngest group had highly impaired scores in the “Treatment” domain. HRQoL was not affected by the presence of FVIII inhibitors.
21	Furuichi et al. [47]/2020/Japan/Peer-reviewed journal article	KIDSCREEN-52	Patient self-report and parent/caregiver report	Patient-reported “physical well-being” score: Age 8-12 y: 55.60 ± 12.10 Age 13-18 y: 50.70 ± 13.80	HRQoL was compared between children with and without hemophilia (control group), with no comparison regarding treatment options. A significant difference was observed between children (aged 8-12 y) in the hemophilia group and those in the control group in 1 dimension of the self-reported HRQoL: the hemophilia group showed a significantly lower mean score for “moods and emotions” (48.20) than the control group (52.40) (P = .023). In contrast, no significant difference was observed in any of the self-assessed HRQoL dimensions between these groups in the adolescent category (13-18 y of age). Alternatively, parent-reported HRQoL differed significantly between patients and controls in several dimensions. In particular, in the lower age category (8-12 y of age), the hemophilia parents’ group had significantly lower scores than the parents’ control group for “moods and emotions” (43.40 and 49.60, respectively; P = .042), “social support and peers” (47.60 and 54.00; P = .041), “self-perception” (45.10 and 50.30; P = .026), and “school environment” (49.00 and 54.60; P = .048). However, no significant differences in any of these dimensions were noted in the records of parents of adolescents.
22	Broderick et al. [48]/2010/Australia/Peer-reviewed journal article	Haemo-QoL I (4-7 y), II (8-12 y), and III (13-16 y)	Patient self-report	Mean Haemo-QoL score: 22.00 ± 10.00 (ages 6-17 y)	QoL was generally high. There was no correlation between QoL and age (r = −0.01) or fitness (r = −0.26) in Australian boys with hemophilia. QoL did not differ between boys with mild, moderate, or severe hemophilia (18.5%, 22.6%, 22.2%, P = .560).
23	Zhang et al. [49]/2019/China/Peer-reviewed journal article	CHO-KLAT	Patient self-report and parent/caregiver report	Child self-report (n = 12): - baseline: 60.69 ± 20.28 - 4-year follow-up: 64.69 ± 13.71 Parent proxy (n = 42): - baseline: 61.01 ± 12.14 - 4-year follow-up: 65.33 ± 15.78	There were no significant differences between the CHO-KLAT scores of 12 proxy and child self-reports (P = .440 and P = .275, respectively). CHO-KLAT scores were higher at follow-up than at baseline, but no statistical significance was observed. Hemophilia decreased HRQoL in patients, but this effect weakened at year 4 compared with baseline (primary outcome). In addition, HRQoL was influenced by bleeding rates, physical activity restriction, financial burden, and treatment (secondary exploratory outcomes). Prophylactic treatment was a key factor contributing to event-free survivor prognosis and optimal therapy for persons with hemophilia.
24	Gringeri et al. [50]/2011/Italy/Peer-reviewed journal article	Haemo-QoL I (4-7 y), II (8-12 y), and III (13-16 y)	Patient self-report and parent/caregiver report	Mean total Haemo-QoL score (all patients): - patient-reported: 29.97 ± 9.30 - parent-reported: 28.70 ± 11.20	A significant difference was found between children and adolescents on episodic treatment vs prophylaxis for the “Family” domain (P < .029, Student’s t-test), which was more impaired in the episodic treatment group (44.00 ± 22.60) than in the prophylaxis group (11.27 ± 8.70). Moreover, children on episodic treatment felt “often” or “always” more overprotected both by their mother (80.0% vs 11.0%) and father (80.0% vs 20.0%) than children on prophylaxis. In the episodic treatment group, 20.0% of children and adolescents perceived that their parents had “often” or “always” to limit their work or leisure time because of their hemophilia compared with none in the prophylaxis group, whereas only 10% perceived that their parents had only “sometimes” their work or leisure time limited.
25	Lock et al. [51]/2016/The Netherlands/Peer-reviewed journal article	Haemo-QoL I (4-7 y), II (8-12 y), and III (13-16 y)	Parent/ caregiver report	Mean total Haemo-QoL score: - baseline: 18.00 (range, 13-23) - post-intervention: 17.00 (range, 12-26)	Disease-specific QoL improved in 3 domains after intervention (nurse home visits): “Family” (P = .040), “Friends” (P = .030), and “Perceived support” (P = .030). No change was reported in other Haemo-QoL domains.
26	Papagianni et al. [52]/2016/Greece/Conference abstract	Haemo-QoL I (4-7 y), II (8-12 y), and III (13-16 y)	Patient self-report and parent/caregiver report	Mean Haemo-QoL scores: - Child self-report Age 4-7 y: 34.02 Age 8-12 y: 26.43 Age 13-16 y: 28.48 (no dispersion data provided) - Parent proxy Age 4-7 y: 38.72 Age 8-12 y: 29.93 Age 13-16 y: 32.41 (no dispersion data provided)	HRQoL scores were widely below 40 for children and below 50 for their caregivers in all dimensions. “Family” and “Treatment” had the highest scores (mean scores of 57.70 and 46.20, respectively), suggesting reduced QoL in these dimensions in children aged 4-7 y. The most impaired Haemo-QoL domain in children aged 8-12 y was “Perceived support” (mean score of 40.63), whereas older children showed greater impairment in the “Family” and “Treatment” domains, but much less than that of younger children (mean score of 36.10 in both domains). The least impaired domains were “Self-view,” “Other persons,” and “Relationships” in the 3 groups. Among Haemo-QoL subscales, “Perceived support” in the age group 8-12 y and “Sports/school” and “Relationships” in the adolescent group were strongly associated with the number of hemarthroses over the previous 12 mos.
27	Hassab et al. [53]/2016/Egypt/Conference abstract	Haemo-QoL I (4-7 y), II (8-12 y), and III (13-16 y)	Patient self-report and parent/caregiver report	Mean “Physical health” Haemo-QoL scores: Age 4-7 y: 62.50 ± 24.02 Age 8-12 y: 70.93 ± 11.94 Age 13-16 y: 76.43 ± 12.69	There was no significant difference in child or parent Haemo-QoL scores between the age groups (P = .200). Forty-four patients (88.0%) had different degrees of anemia. The degree of anemia was significantly correlated with child Haemo-QoL scores (r = 0.29, P = .040), mainly with “View” and “School” domain scores. Both child and parent Haemo-QoL scores were significantly correlated with the factor activity level, disease duration, duration of joint disease, number of bleeding events in the past year, and number of joints affected. Five patients (10.0%) underwent synovectomy. The Haemo-QoL score was significantly lower after intervention (P = .043), especially in the “Physical health,” “Feeling,” “View,” “Family,” “School and sports,” “Treatment,” and “Dealing” domains.
28	Khair et al. [54]/2012/United Kingdom/Peer-reviewed journal article	KINDL Haemo-QoL I (4-7 y), II (8-12 y), and III (13-16 y)	Patient self-report	Total KINDL scores: Age 6-7: 77.61 ± 14.20 Age 8-12: 70.40 ± 8.90 Age 13-17: 70.38 ± 12.30 Total Haemo-QoL scores: Age 6-7: 28.09 ± 14.80 Age 8-12: 41.43 ± 10.50 Age 13-17: 24.44 ± 12.70	HRQoL in children was generally good, with the highest impairments in boys aged 8-12 y. Boys aged 8-16 y reported good physical performance (80.00 ± 16.00), with the highest impairments in the “endurance” and “mobility” domains. Boys who engaged in sports had significant better physical performance and HRQoL than boys who did not. Sedentary lifestyle had a negative impact on subjective physical performance and number of days lost of children.
29	Poon et al. [42]/2012 (HUGS-Va)/ United States/Peer-reviewed journal article	PedsQL	Patient self-report and parent/caregiver report	Mean total PedsQL score: 85.90 ± 13.80	Among children, PedsQL scores generally decreased with increasing disease severity, and no significant differences were found between persons with severe hemophilia receiving prophylaxis (mean total PedsQL: 84.10 ± 14.20) and on-demand treatment (mean total PedsQL: 86.50 ± 12.40). In children, the mean total PedsQL scores as well as mean scores across all PedsQL subscales decreased as the severity of joint pain increased, with statistically significant (P < .050) and minimally clinically important differences between groups with no or little joint pain and those with more severe joint pain. With increasing motion limitation, mean total PedsQL as well as all PedsQL subscale scores also decreased. Statistically significant (P < .050) and clinically important differences were also observed in the total PedsQL, physical functioning, psychosocial health, and social functioning scores.

CBD, congenital bleeding disorder; FVIII, coagulation factor VIII; HRQoL, health-related quality of life; QoL, quality of life; VAS, visual analog scale; WFH, World Federation of Hemophilia.

a Self-report includes data collection by the physician interviews for younger children (usually aged ≤7 y).

b Overall score or general physical health score if overall score is unavailable.

**Figure 2:**
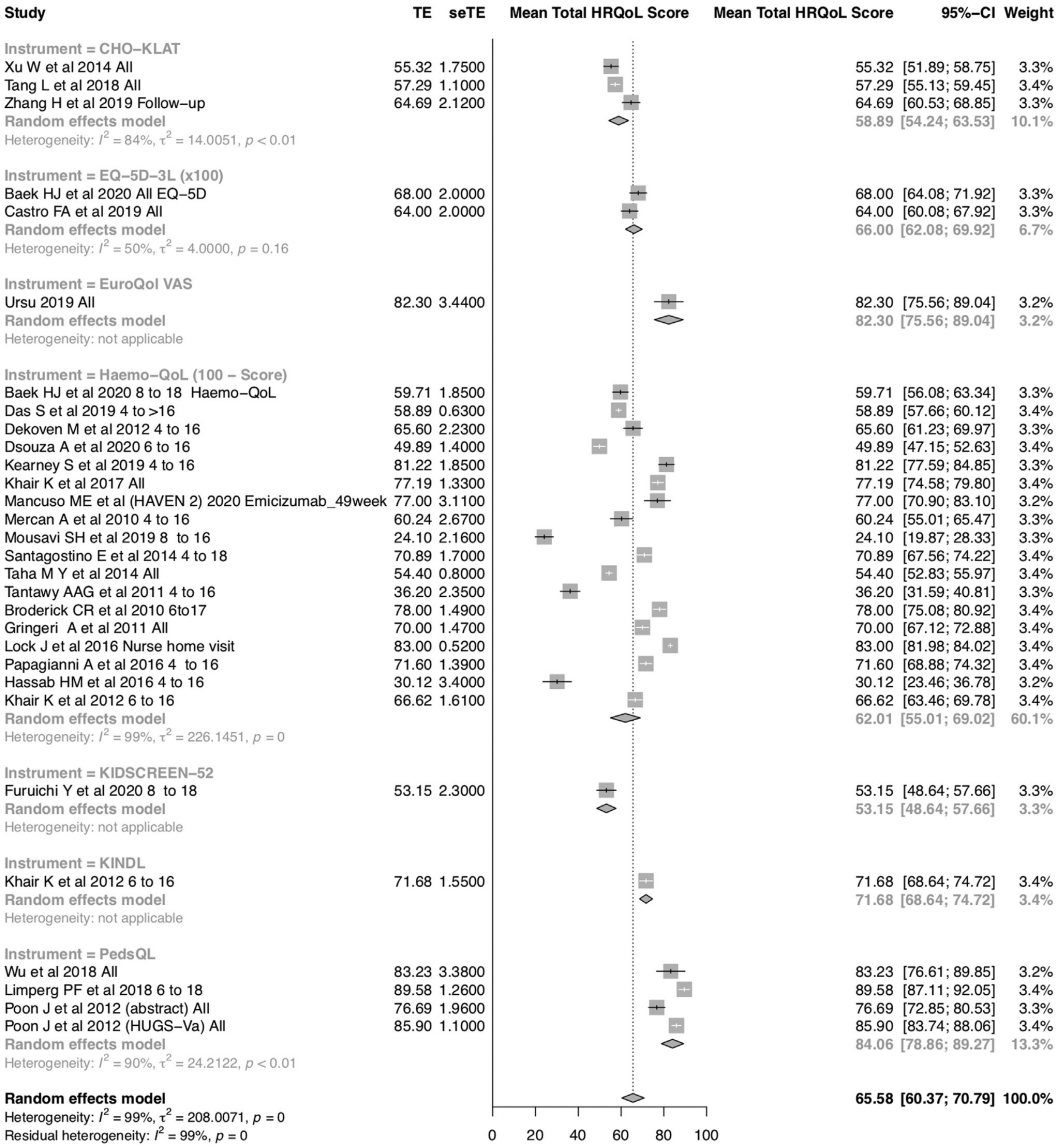
Meta-analysis of total HRQOL scores according to QOL assessment instruments. Higher scores indicate better HRQOL (range, 0 to 100). To match the amplitude and effect direction of most instruments, EQ-5D-3L scores are presented as 100 times the original figures and Haemo-QoL scores are presented as 100 minus the original scores because, originally, higher scores indicated worse QOL in the Haemo-QoL questionnaire. All other scores are presented on the original scale of each specific instrument. TE, treatment effect; seTE, standard error for treatment effect; HRQoL, health-related quality of life; CI, confidence interval.

The subgroup meta-analysis according to specific HRQoL assessment instruments demonstrated that high heterogeneity persisted even among studies that used the same instrument to assess HRQoL. Figure 2 shows the total and subgroup meta-analyses for the total HRQoL scores by the type of instrument.

Additional subgroup meta-analyses according to specific age groups (0-7, 8-12, and 13-18 years) and inhibitor status were unable to explain the high heterogeneity (Supplementary Figs. 1 and 2). A comparative meta-analysis of the 2 studies that provided sufficient information to directly compare the mean total HRQoL scores, as measured with EQ-5D-3L and PedsQL, between patients with and without FVIII inhibitors indicated a statistically significant increase in the SMD of HRQoL scores favoring patients without FVIII inhibitors (Supplementary Fig. 3; SMD = 0.57; 95% CI, 0.18-0.97; *I*^2^ = 0.0%) [32,40].

**Figure 3:**
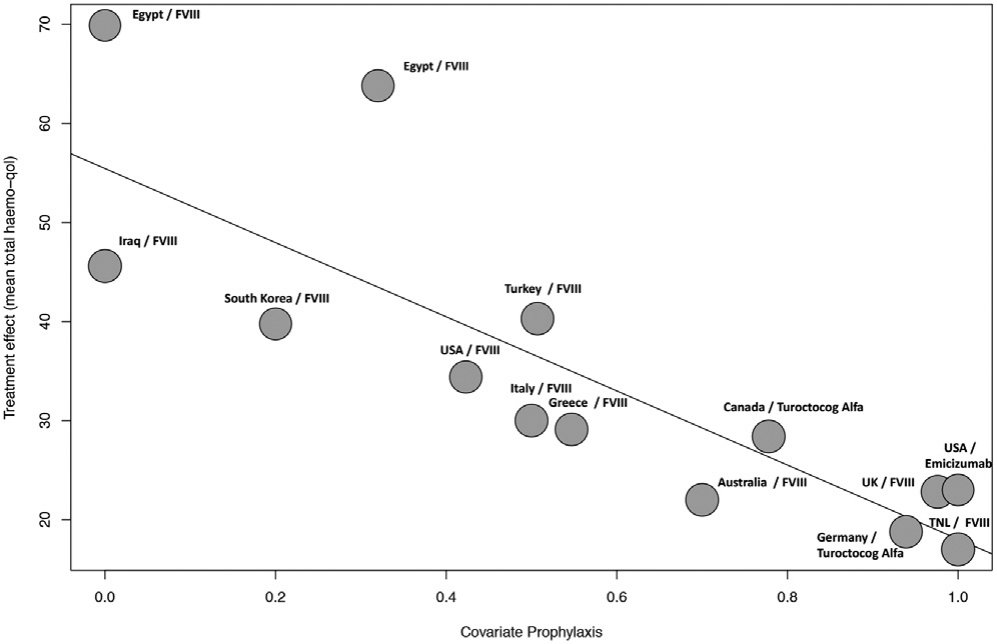
Meta-regression analysis of the impact of the proportion of patients receiving active prophylactic treatment for hemophilia A on total quality of life scores as measured with the Haemo-QoL questionnaire. Higher scores indicate worse HRQOL. Mixed-effects model, *τ^2^* = 42.7323 (SE = 23.9699), *I^2^* = 94.67% (total heterogeneity), *H^2^* = 18.76% (unaccounted heterogeneity), *R^2^* = 79.34% (amount of heterogeneity accounted for). y = 55.4442−37.4145 × x. HRQOL, health-related quality of life; FVIII, coagulation factor VIII; USA, United States of America; UK, United Kingdom; TNL, The Netherlands.

We performed a meta-regression analysis to assess the impact of the proportion of patients receiving active prophylaxis for hemophilia A in each study on the total QoL scores as measured with the Haemo-QoL questionnaire, considering its original scoring system of higher scores indicating worse QoL. This analysis included 14 studies from 12 countries reporting a range of 0.0% to 100% of patients on a prophylactic regimen with regular administration of FVIII (11 studies), turoctocog alfa (2 studies), or emicizumab (1 study). There was a significant negative association between higher Haemo-QoL scores (indicating worse QoL) and the proportion of patients receiving continuous prophylactic treatment, ie, the higher the proportion of patients receiving effective prophylactic treatment, the lower the mean total Haemo-QoL score, indicating better HRQoL. This analysis demonstrated that 79.3% (*R*^*2*^) of the observed 94.7% total heterogeneity (*I*^*2*^) was explained by the proportion of patients receiving effective prophylactic treatment. The meta-regression analysis also demonstrated that 3 of the 6 studies with the highest HRQoL scores (mean total Haemo-QoL score of <30) were treated with hemostatic agents such as turoctocog alfa (in 2 studies) and emicizumab (in 1 study) (Figure 3).

The KIDSCREEN-52 and CHO-KLAT were the most comprehensive tools among all identified instruments. They covered, respectively, 11 and 9 of the 15 domain categories that were mapped from the 6 QoL assessment instruments used in the included studies. A comparison of the domains assessed in the QoL instruments used in the 29 included studies is shown in Table 4 [[1], [2], [3], [4], [5], [6], [7], [8], [9], [10], [11], [12], [13], [14], [15], [16], [17], [18], [19], [20], [21], [22], [23], [24], [25], [26],^31^,^37^,^42^].

**Table 4:** Comparison of instruments used in studies assessing the QoL of children with hemophilia.

QoL assessment tool	PedsQL	EQ-5D-3L/EuroQol VAS	Haemo-QoL	CHO-KLAT	KIDSCREEN-52	KINDL
Type of instrument	Generic, children	Generic, children and adults	Hemophilia-related QoL, including long and short versions for ages 4-7, 8-12, and 13-16 y	Hemophilia-related QoL, age 4-18 y	Generic, pediatrics	Generic, pediatrics
No. of studies	5 [2,4,15,16,42]	3 [1,5,6]	17 [5,[7], [8], [9], [10], [11], [12], [13], [14],^17^,^18^,^20^,^22^,[24], [25], [26],^31^]	3 [3,19,23]	1 [21]	1 [37]
Physical health/pain/discomfort/mobility	Yes	Yes	Yes	Yes	Yes	Yes
Emotional functioning/ability to deal with, fear, anxiety, frustration/feeling	Yes	Yes	Yes	Yes	Yes	Yes
Social functioning/family/friends	Yes	No	Yes	Yes	Yes	Yes
School functioning	Yes	No	Yes	No	Yes	Yes
Psychosocial health	Yes	No	Yes	Yes	Yes	Yes
Self-image/view	Yes	No	No	Yes	Yes	Yes
Treatment quality/satisfaction and acceptance of treatment	No	No	Yes	Yes	No	No
Autonomy/independence/activities of daily living/self-care	No	Yes	No	Yes	Yes	No
Disease education/knowledge/understanding	No	No	No	Yes	No	No
Report of injuries to family members/recognition and control of symptoms	No	No	No	Yes	No	No
Perceived support	No	No	No	No	Yes	No
Feeling different from others/attitude and behavior of others	No	No	No	No	Yes	No
Sports and leisure	No	No	No	No	Yes	No
Relationship/partnership	No	No	No	No	No	No
Financial resources	No	No	No	No	Yes	No

QoL, quality of life.

Regarding the QoL domains that most influenced the overall QoL assessment, the domains “View,” “Others,” and “Relationships” had the highest QoL scores for the age groups 0 to 7 years, 8 to 12 years, and 13 to 18 years, respectively. The domains “Family” (overprotection) and “Perceived support” had the lowest QoL scores for the age groups 0 to 7 years and 8 to 18 years, respectively. Table 5 shows the frequency of the highest and lowest domain scores per age group among studies reporting HRQoL in children and adolescents with hemophilia A.

**Table 5:** Frequency of the highest and lowest scored domains per age group among studies reporting HRQoL in children and adolescents with hemophilia A.

0-7 y age group^a^				8-12 y age group^b^				13-18 y age group^c^			
Top domains per study indicating better QoL^a^		Top domains per study indicating worse QoL^a^		Top domains per study indicating better QoL^a^		Top domains per study indicating worse QoL^a^		Top domains per study indicating better QoL^a^		Top domains per study indicating worse QoL^a^	
View	7 (50.0%)	Family	9 (64.0%)	Others	5 (33.3%)	Support	4 (26.7%)	Relationships	3 (21.4%)	Support	4 (30.8%)
Physical health	4 (28.6%)	Emotional	1 (7.0%)	Treatment	3 (20.0%)	Sports	4 (26.7%)	Dealing	2 (14.3%)	Sports	3 (23.1%)
Feeling	2 (14.3%)	View	1 (7.0%)	Physical	2 (13.3%)	School	4 (26.7%)	Feeling	2 (14.3%)	School	3 (23.1%)
Sports	1 (7.1%)	Friends	1 (7.0%)	Friends	2 (13.3%)	Family	2 (13.3%)	Others	2 (14.3%)	Physical	2 (15.4%)
School	1 (7.1%)	Support	1 (7.0%)	Social	1 (6.7%)	Physical	2 (13.3%)	Treatment	2 (14.3%)	Family	1 (7.7%)
—	—	Sports	1 (7.0%)	Feeling	1 (6.7%)	Dealing	1 (6.7%)	Physical	1 (7.1%)	Friend	1 (7.7%)
—	—	School	1 (7.0%)	—	—	Financial	1 (6.7%)	View	1 (7.1%)	Self-perception	1 (7.7%)
—	—	—	—	—	—	—	—	—	—	Treatment	1 (7.7%)

Dealing: related to the recognition and control of symptoms and acceptance of disease (Haemo-QoL). Emotional: problems with emotional functioning (PedsQL; KINDL). Family: related to the level of overprotection from parents and impact of hemophilia on family life (Haemo-QoL; KIDSCREEN-52; KINDL). Feeling: related to emotional well-being, including feeling worried, sad, lonely, etc., due to hemophilia (Haemo-QoL). Financial: related to perception relative to friends of having enough financial resources for expenses (KIDSCREEN-52). Friends: related to relationship with friends and ability to talk with them about hemophilia (Haemo-QoL; KINDL). Others: related to feeling different from others and the attitude and behavior of others (Haemo-QoL). Physical health: related to the level of joint pain and other issues related to physical health (Haemo-QoL; PedsQL ; KIDSCREEN-52; KINDL). Relationships: related to romantic partnership due to hemophilia (Haemo-QoL). Self-perception: related to satisfaction and worries with patient’s own body, clothes, and general appearance (KIDSCREEN-52). Social: related to problems with social functioning (Haemo-QoL; PedsQL). School: related to participating in different types of physical and leisure activities and intellectual activities inside School (Haemo-QoL; PedsQL; KINDL). Sports: related to participating in different types of physical and leisure activities outside School (Haemo-QoL). Support (perceived support): related to consideration and understanding from others in relation to hemophilia (Haemo-QoL). Treatment: related to the satisfaction with and acceptance of the treatment, health care management, and injection-related constraints (Haemo-QoL). View: related to the attitude toward others and the impact of hemophilia on ability to do things (Haemo-QoL).Note: none of the 3 studies that used the CHO-KLAT instrument reported detailed results per instrument domain.

HRQoL, health-related quality of life; QoL, quality of life.

a Fourteen subgroups from 13 studies (considering both arms from the randomized trial by Gringeri et al. [50]); 268 patients assessed in 11 countries.

b Fifteen subgroups; 534 patients assessed in 13 countries.

c Thirteen subgroups from 15 studies; 334 patients assessed in 11 countries.

Publication bias assessment revealed a highly heterogeneous yet fairly balanced distribution of data points in the funnel plot for the 14 studies that used the Haemo-QoL questionnaire (Supplementary Fig. 4). Begg’s rank correlation test results indicated a low likelihood of publication bias (*P* value for asymmetry in the funnel plot = .154).

## 4 Discussion

To the best of our knowledge, this is the most comprehensive systematic review of HRQoL assessment in children and adolescents with hemophilia A conducted to date. Two recent reviews evaluating HRQoL among children and adolescents with hemophilia A presented distinct scopes: one focused on the comparative effectiveness of hemophilia A treatments and the other was not a hemophilia-specific review [56,57]. In addition, the last systematic review that assessed HRQoL questionnaires for children and adolescents with hemophilia A was published in 2017 [5]. Therefore, the present systematic review represents a necessary update.

In general, studies of moderate quality were considered. The risk of bias ranged from low to moderate, and the risk of publication bias was considered to be low.

The main finding of this study is that HRQoL in children and adolescents with hemophilia A is strongly influenced by an effective prophylactic treatment, as demonstrated by our meta-regression analysis. Initially, because we identified high heterogeneity among studies in the results for the mean total HRQoL scores in children and adolescents with hemophilia A, we performed a random-effects meta-analysis of our primary outcome (mean total HRQoL score) by the type of instrument. However, the summary measure could not be considered representative of the effect size of the set of studies, and the investigation of possible causes of heterogeneity was therefore the focus of the statistical analysis. The high heterogeneity could not be adequately explained by the difference in the instruments applied, by the difference in age groups, or by the difference in the proportion of patients without or with FVIII inhibitors in each study. However, the meta-regression analysis of the effect of the proportion of patients receiving effective prophylactic treatment with regular administration of FVIII, turoctocog alfa, or emicizumab was able to identify a statistically significant association between a higher proportion of patients on effective prophylaxis and better QoL, represented by lower mean total scores in the Haemo-QoL questionnaire. These differences in the proportion of patients on effective prophylactic treatment are likely to reflect inequalities in access to care across the globe with a corresponding impact on the QoL.

Only 18.8% residual heterogeneity could not be adequately explained by the meta-regression analysis. It should be noted that studies reporting better QoL (mean total Haemo-QoL score of <30) had a higher proportion of patients treated with new hemostatic agents, such as turoctocog alfa (extended half-life FVIII) and emicizumab (monoclonal antibody). This indicates that the best results in terms of QoL for the pediatric population can be obtained using more convenient therapeutic agents associated with care settings with strong clinical support (studies with turoctocog alfa and emicizumab were conducted in Germany, Canada, and United States).

A relevant finding is the limited number of instruments that have been used to measure QoL in children and adolescents with hemophilia. We identified only 6 distinct instruments in the 29 included studies. Another interesting finding is the predominant use of Haemo-QoL in the pediatric population because this questionnaire was used in more than half of the included studies. Reasons for the preference for this instrument include the availability of specific versions for different age groups, the relative simplicity of application, the availability of versions for child self-report and parent/caregiver proxy-report, and easy access to translated versions in variety of languages.

Assessment of risk of bias in the present review was challenging because we identified 4 distinct study designs, which included noncomparative cross-sectional and cohort studies, comparative cohort studies, and a randomized controlled trial. There is no consensus in the literature regarding the best instruments for assessing the methodological quality of noncomparative cross-sectional or cohort studies. We chose to use NOS for cohort studies and NOS adapted for cross-sectional studies, with exclusion of domains referring to the assessment of the comparative arm.

Although only the comparative meta-analysis between patients with and without inhibitors for the outcome “SMD of HRQoL scores” was considered technically appropriate (*I*^2^ = 0.0% between the 2 included studies), the results of individual studies provide relevant information for the comparative assessment of HRQoL among other subgroups of patients. Some particularly noteworthy findings from individual studies are as follows:

• HRQoL tends to be better in children and adolescents with hemophilia A than in adults with the disease [1,13,14].
• Pain associated with joint bleeding seems to be the most important variable in explaining the reduction in perceived QoL related to physical health [2,5,23,26,42].
• HRQoL estimates obtained from parents/caregivers tend to be closely related to those obtained directly from children and adolescents [3,19,23,31].
• Sports and school attendance are positively associated with QoL [4].
• QoL of children and adolescents with hemophilia receiving prophylactic treatment in high-quality care settings appears to be comparable with that of children and adolescents without hemophilia; however, this finding should be regarded with caution because of a possible “disability paradox” that has been described in this population [4,58].
• There is a trend toward deterioration in QoL with increasing age among children and adolescents [8].
• Children and adolescents with moderate-to-severe hemophilia A consider “Family” (associated with parental overprotection) one of the domains that most negatively impact their HRQoL [34,35,39,50,52].
• A lower proportion of patients receiving prophylactic treatment appears to be associated with worse HRQoL in the “Family” domain (overprotection) [4,36,48].
• Children and adolescents receiving on-demand treatment report feeling overly protected by their parents and with greater restriction of physical activity than those receiving prophylactic treatment [52].
• Treatment with new hemostatic agents, such as turoctocog alfa (extended half-life FVIII) and emicizumab (monoclonal antibody), is associated with further improvement of HRQoL compared with baseline HRQoL [38,43,46].
• Patients who participate in sports have significantly better QoL than those who do not [37].

The limitations of this systematic review should also be considered. First, the high heterogeneity among studies prevented us from reliably providing a useful summary effect size estimate. Second, although the meta-regression analysis was able to explain much of the heterogeneity among studies, significant inconsistencies persisted. The assessment of the risk of bias of noncomparative studies was limited by the use of adapted scales without previous validation for this purpose. Finally, the search strategy used in the current systematic review focused on terms for QoL. We did not search for studies reporting QoL outcomes associated with specific products or treatment regimens. We acknowledge that studies reporting QoL measured in the context of clinical trials of specific hemophilia A treatment products are misrepresented in the final selection of studies. On the other hand, our search strategy leaned toward the identification of real life cross-sectional and cohort studies that reported QoL in diverse accessibility of care settings. Despite these limitations, the present systematic review provides a comprehensive summary of published research on HRQoL in children and adolescents with hemophilia A over a 10-year period.

## 5 Conclusions

There is substantial clinical and methodological heterogeneity among studies evaluating the QoL of children and adolescents with hemophilia A. This limits the conclusions that can be drawn from the current systematic review and meta-analysis. A more standardized and homogeneous approach to assess QoL in this patient population is needed. A high proportion of patients receiving prophylactic treatment in high-quality care settings and the use of new hemostatic agents with extended half-life are factors associated with better mean total HRQoL scores.
