# Supplementary material for: Quality of life in children and adolescents with hemophilia A: A systematic review and meta-analysis
Source: Res Pract Thromb Haemost. 2022 Dec 9;7(1):100008. doi: 10.1016/j.rpth.2022.100008 (PMC10031335; doi:10.1016/j.rpth.2022.100008)
Supplement: Supplementary material [file mmc1.docx]

**Supplementary Table 1**. Complete search strategy used for MEDLINE (PubMed) and for Cochrane Library (Wiley). Equivalent strategies were used for EMBASE and LILACS.

| Database | Search Strategy |
| --- | --- |
| PubMed (MEDLINE) | ("Hemophilia A"[Mesh] OR "Hemophilia B"[Mesh] OR “Hemophilia As” OR “Hemophilia” OR “Congenital Hemophilia A” OR “Congenital Hemophilia As” OR “Classic Hemophilia” OR “Classic Hemophilias” OR “Haemophilia” OR “Autosomal Hemophilia A” OR “Autosomal Hemophilia As” OR “Factor VIII Deficiency” OR “Entry Terms:” OR “Hemophilia Bs” OR “Factor IX Deficiencies” OR “Factor IX Deficiency” OR “Hemophilia B Leyden” OR “Hemophilia B(M)” OR “Hemophilia Bs (M)” OR “Plasma Thromboplastin Component Deficiency” OR “F9 Deficiency” OR “F9 Deficiencies” OR “Christmas Disease” OR “Haemophilia B” OR “Haemophilia Bs”)  AND  (("Quality-Adjusted Life Years"[Mesh] OR "Quality-Adjusted Life Years" OR “Quality-Adjusted Life Year” OR “Quality Adjusted Life Years” OR “QALY” OR “Quality Adjusted Life Year” OR “Healthy Years Equivalents” OR “Healthy Years Equivalent” OR “Adjusted Life Years” OR “Adjusted Life Year”) OR ("Quality of Life"[Mesh] OR "Quality of Life" OR “Life Quality” OR “Health-Related Quality Of Life” OR “Health Related Quality Of Life” OR “HRQOL”) OR (“utility” OR “utilities” OR “disutility” OR “disutilities” OR “QoL” OR “DALY” OR “disability-adjusted life years” OR “disability adjusted life years”)) |

**Supplementary Figure S1**. Subgroup meta-analysis according to specific age groups.

**Supplementary Figure S2**. Subgroup meta-analysis according to inhibitor status.

**Supplementary Figure S3**. Meta-analysis of studies that compared mean total HRQOL scores between patients with and without factor VIII inhibitors (standardized mean difference).

**Supplementary Figure S4**. Funnel plot of the 14 studies that used the Haemo-QoL questionnaire.
